# Analytical Validation of *SOD2* Genotyping

**DOI:** 10.3390/mps6010004

**Published:** 2022-12-31

**Authors:** Marija Debeljak, Stacy Riel, Ming-Tseh Lin, James R. Eshleman, Channing J. Paller

**Affiliations:** 1 Department of Pathology, The Sol Goldman Pancreatic Cancer Research Center, Johns Hopkins University School of Medicine, Baltimore, MD 21287, USA; 2 Department of Oncology, The Sol Goldman Pancreatic Cancer Research Center, Johns Hopkins University School of Medicine, Baltimore, MD 21287, USA; 3 The Sidney Kimmel Comprehensive Cancer Center, Johns Hopkins University School of Medicine, Baltimore, MD 21231, USA

**Keywords:** *SOD2* gene, pyrosequencing, prostate cancer, prostate-specific antigen (PSA)

## Abstract

Manganese superoxide dismutase-2 (*SOD2*) plays a crucial role in cells’ protection against mitochondrial oxidative damage. A genetic polymorphism in the mitochondrial targeting sequence of the *SOD2* gene has been implicated in various diseases, including prostate cancer. Paller et al. have shown an increase in prostate-specific antigen (PSA) doubling time in patients with the Ala/Ala (wildtype) genotype when treated with pomegranate/grape extract antioxidants. We developed and validated a pyrosequencing assay that detects the common germline *SOD2* SNP (rs_4880) with the aim of identifying men with castrate-resistant prostate cancer eligible for an antioxidant therapy clinical trial. We first selected 37 samples from the 1000 genomes study with known genotypes determined using Illumina-based sequencing and confirmed them by Sanger sequencing. In a blinded design, we then performed the new pyrosequencing assay on these samples and assigned genotypes. Genotypes for all 37 samples (13 homozygous Ala, 12 heterozygous Ala/Val, and 12 homozygous Val) were all concordant by pyrosequencing. The pyrosequencing assay has been live since May 2018 and has proven to be robust and accurate.

## 1. Introduction

Biochemically recurrent (BCR) patients experiencing rising prostate-specific antigen (PSA) following local therapy (prostatectomy and/or external beam radiation) make up the largest subset of prostate cancer patients undergoing monitoring and treatment in the United States. The standard of care for these patients ranges from watchful waiting to androgen-deprivation treatment (ADT), but many of them seek treatment alternatives to ADT because of its significant impact on quality of life, including potential obesity, erectile dysfunction, hot flashes, decreased muscle mass, and osteoporosis. Consequently, the use of complementary medical products is widespread among prostate cancer patients, with 39% of recently diagnosed patients and 51% of patients overall reporting that they have used such products as herbal, vitamin, and mineral dietary supplements (primarily antioxidants) to try to boost their immune system and prevent disease progression [1,2,3,4,5,6]. 

Superoxide dismutases (SODs) are antioxidant enzymes and there are three *SOD* genes that have evolved in mammals: manganese superoxide dismutase (MnSOD) in mitochondria, copper-zinc superoxide dismutase (Cu-Zn SOD) in the cytosol, and extracellular superoxide dismutase (EC-SOD) in the extracellular compartments [7]. MnSOD, encoded by the *SOD2* gene, has a scavenging role during oxidative phosphorylation, where it converts superoxide anion into oxygen and hydrogen peroxide [7,8,9]. The critical importance of this activity has been demonstrated by independent studies that showed that deletion of the mouse *Sod2* gene was incompatible with life [10,11].

Signal sequences at the N-terminus target proteins to their respective compartments within the cell [12,13,14]. A single amino acid polymorphism in the mitochondrial targeting sequence of the human mitochondrial *SOD2* gene that encodes for valine (Val) instead of alanine (Ala) at codon 16 has been shown to be less efficiently imported into mitochondria [15,16,17,18,19]. As a consequence, individuals with Val/Val or Ala/Val genotypes were shown to have less active MnSOD [15], although this remains to be confirmed in an independent laboratory. Interestingly, the allele frequency for Ala is relatively common in the Caucasian (49%) and African American (41%–45%) populations, while homozygous Ala is present in only 10%–20% of Asians, and the Val allele is more frequent (80%) in the Japanese population [16,20,21,22].

Identifying biomarkers that will predict response to antioxidant therapies could potentially allow some patients to improve their quality of life and help reduce morbidity. The rationale to further explore this *SOD2* biomarker is supported by three studies. Muscadine grape skin and pomegranate trials showed a 6–12 month lengthening of PSA doubling time (PSADT) in the BCR AA-*SOD2* population but not in the control group or in VV or VA patients [23]. Similarly, the Physician’s Health Study found that men in a broader population with the AA-*SOD2* genotype randomly assigned to β-carotene (antioxidant) treatment (versus placebo) had a relative risk of 0.6 (95% CI, 0.2–0.9) for fatal prostate cancer, but found no significant association in men with the VV/VA genotypes [8].

In order to test the hypothesis that identifying biomarkers will predict response to antioxidant therapies, a SOD2 test was validated in a Clinical Laboratory Improvement Amendments (CLIA)-certified molecular laboratory and was developed for a phase III trial to determine whether 48 weeks of antioxidant treatment compared with placebo significantly lengthens PSADT in AA-SOD2 BCR men. The trial, which is called Muscadine Plus (MPX) in Men with Prostate Cancer (NCT03535675), is an 80-patient, phase III, randomized, double-blind clinical trial was being performed at 15 sites in the United States and using muscadine grape skin extract. Unfortunately, the trial closed for futility 1 July 2022, as it would not meet its primary endpoint. An analysis is underway to see if any of the biomarker endpoints were significant. The CLIA test is also being used in two other diet-based trials, one weight loss intervention in breast cancer (NCT04499950) and another plant-based diet in prostate cancer (NCT05471414), to see if genotype correlates with outcomes.

We report the analytic validation of the codon 16 polymorphism in the mitochondrial leader sequence of the *SOD2* gene using pyrosequencing. After the pyrosequencing is performed, a given patient’s result is pattern-matched to the three pyrograms representing each of the genotypes. We selected 13 homozygous wildtype (WT), 12 heterozygous, and 12 homozygous variant samples with established genotypes from the 1000 genomes project, confirmed their genotypes using Sanger sequencing, and used them for orthogonal validation of the pyrosequencing assay. The assay has been live since May 2018, and approximately 120 runs were completed to select 80 men with castrate-resistant metastatic prostate cancer with the Ala/Ala genotype to be eligible for the clinical trial.

## 2. Materials and Methods

### 2.1. Samples

We obtained 37 DNA samples from the NIGMS Human Genetic Cell Repository at the Coriell Institute for Medical Research (Camden, NJ, USA) under a research agreement with the 1000 Genomes Consortium (See Table 1). Samples were chosen based on their genotypes and included a total of 13 Ala/Ala wildtype homozygotes, 12 Ala/Val heterozygotes, and 12 Val/Val variant homozygotes. 

### 2.2. Primer Design and Sanger Sequencing Confirmation

Based on previous findings, *SOD2* SNP analysis was restricted to a small region around codon 16 within exon 2 (NM_ NM_000636.4(SOD2):c.47T>C (p.Val16Ala)). Five different forward primers and two different reverse primers were designed using Primer3 (https://bioinfo.ut.ee/primer3-0.4.0/, accessed on 9 November 2022). All primers were ordered from IDT Technologies (Coralville, IA) with M13 forward and M13 reverse universal primers. The best-performing primers (used in the final clinical protocol) were as follows: forward primer 5′-tgtaaaacgacggccagt-ACTGACCGGGCTGTGCTT-3′ (SOD2 region, upper case letters and underlined) and reverse primer 5′-biotin-caggaaacagctatgacc-GCGTTGATGTGAGGTTCCAG-3′, (*SOD2* region, upper case and underlined), where M13 forward and reverse universal primers are indicated in lower case letters. Samples were PCR amplified using 100 ng genomic DNA. Cycling conditions were as follows: 96 °C for 1 min; 35 cycles of 95 °C for 30 s; 62 °C for 30 s; 72 °C for 1 min, and a final extension of 72 °C for 10 min. The expected size of the full-length PCR product was 174 bp, including the M13 primers. After amplification, PCR products were visualized by gel electrophoresis (Novex TBE Gels, Life Technologies, Waltham, MA, USA) and Sanger sequenced to confirm the previously determined genotypes. 

### 2.3. Pyrosequencing

PCR-amplified products were generated using the same primers optimized for Sanger sequencing above, except that the reverse primer was biotinylated at the 5′ end to capture the bottom (anti-sense) strand for pyrosequencing. Each reaction contained 1X PCR buffer, 1.5 mmol/L MgCl_2_, 0.2 mmol/L of each dNTP, 5 pmol of forward primer (listed above), 5 pmol of reverse primer (biotinylated), 0.8 units of HotStar TaqDNA polymerase (Qiagen), 10 ng of template DNA, and dH_2_O to a 25 ul final volume. Pyrosequencing was done on the PyroMark Q24 (Qiagen) instrument, as previously reported, in a reaction containing 0.3 μmol/L sequencing primer (5′- AGCAGGCAGCTGGCTCCG -3′) and annealing buffer. The nucleotide triphosphate dispensation order for codon 16 was G,A,T,A,C,T,A,G,A,T,A,T.

### 2.4. Final Protocol

A total of 3 mL of peripheral blood from each patient was submitted to the Molecular Diagnostics Laboratory at The Johns Hopkins Hospital. DNA was isolated from 350 ul of peripheral blood using the Qiagen EZ1 Advanced XL instrument per the manufacturer’s protocol (Qiagen, Valencia, CA, USA). Isolated DNA was quantified using Qubit 2.0 (Life Technologies, Carlsbad, CA, USA). We identified EBV-transformed lymphoblastoid cell lines to use as controls for each of the three genotypes (homozygous WT = NA10830, heterozygous control = GM12878, and homozygous variant control = NA06991). GM12878 is a commonly used reference sample whose use is advocated by the National Institute of Standards and Technology and the Centers for Disease Control (CDC). DNA samples were PCR amplified and pyrosequenced as described above. Analysis was done using Pyromark Q24 software, and pyrograms were pattern-matched for the genotype. The full protocol is available upon request.

## 3. Results

### 3.1. Samples and Sanger Confirmation of Genotypes

We obtained 37 samples from the 1000 genomes study and determined their genotypes at exon 2 of the *SOD2* gene using the 1000 genomes data sequenced on the Illumina platform. We then tested all possible primer combinations (see Section 2) on a subset of 6 of the 37 samples (indicated by * in Supplemental Table S1). The six-sample subset included two samples for each of the three genotypes (Ala/Ala, GCT/GCT = NA18507 and NA18508; Ala/Val, GCT/GTT = NA07048 and NA18488 G/A; and Val/Val, GTT/GTT = NA18486 and NA18489 A/A). The primer pair that performed best was chosen for the validation study. We then confirmed the genotypes of all 37 samples by cross-referencing with the known results from the 1000 genomes project. We compared our Sanger sequencing results to Illumina sequencing, which demonstrated 100% concordance and served as the consensus genotype (Table 1). 

### 3.2. Pyrosequencing Assay Design

**Figure 1:**
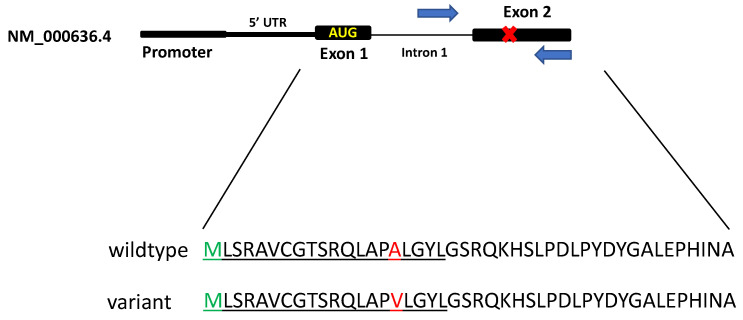
*SOD2* gene overview showing 2 exons out of total of 5 exons. Start codon, AUG (green), is shown in Exon 1. “X” (red) in Exon 2 denotes the polymorphism in codon 16. Blue arrows indicate primer position for PCR amplification. Amino acid sequence for Exon 1 and Exon 2 is listed for both wildtype and variant where start codon, M (green), amino acid polymorphism position (red), and mitochondrial targeting sequence (underlined) are indicated.

**Figure 2:**
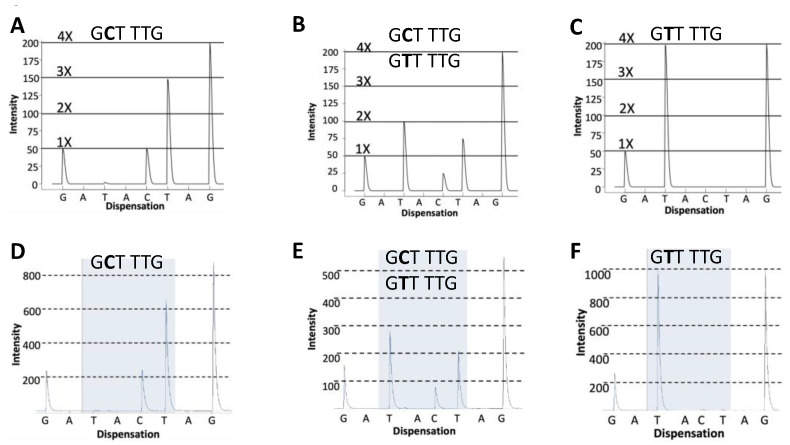
Pyrograms generated in silico using the software Pyromaker, which produces an expected pyrogram based on user input (**A**–**C**) and pyrograms experimentally generated by pyrosequencing (**D**–**F**). Panels (**A**) and (**D**) show pyrograms for homozygous WT, G**C**T/G**C**T (Ala/Ala) genotype. Panels (**B**) and (**E**) show pyrograms for heterozygous genotype, G**C**T/G**T**T (Ala/Val). Panels (**C**) and (**F**) show homozygous variant genotype, G**T**T/G**T**T (Val/Val). The three guanines, which contribute to the final 4G peak in the electropherograms, are not shown in the text.

We initially considered both Sanger sequencing and pyrosequencing for genotyping but chose pyrosequencing because the region of interest was small and the patterns of Ala/Ala, Ala/Val, and Val/Val were predicted to be completely distinct [24]. We designed and tested multiple PCR amplification primers (see Section 2) and four different sequencing primers. One critical aspect of a pyrosequencing assay design is to establish the amount of signal that results from the addition of a single base to both alleles (thereby establishing 1X activity as a “ruler”). It is important to establish this level prior to sequencing into the unknown area of a gene (the codon 16 polymorphism, in this case). We chose the primer (Figure 1) that gave the most robust signal and that established the 1× ruler, which is proportional to the incorporation of one base into both alleles of the elongating DNA strand during a single dispensation [25,26,27]. The dispensation sequence was optimized to maintain the phase of the two alleles irrespective of the genotype (Figure 2).

### 3.3. Accuracy and Precision

Four different samples were included on two to three different pyrosequencing runs to establish inter-run reproducibility. In order to determine intra-run reproducibility, duplicates of four different samples (NA18501, NA18505, NA18949, and NA18499) were included on the same pyrosequencing run. Results were concordant and reproducible (Table 2).

We included four different controls in this assay (homozygous wild type, heterozygous, homozygous variant, and non-template control, NTC). As such, we expected the heterozygous control to be present between 40% and 60%. This range is easily achievable by pyrosequencing technology, which has a limit of detection between 3% and 5%.

We calculated the percent of C:T alleles in all of the samples that were pyrosequenced. The background noise for the homozygous (C/C) wildtype was ≤5% T, and for the homozygous (T/T) variant, it was ≤5% C. The heterozygous (C/T) control was consistently between 45–55% C:T. Non-template controls consistently demonstrated no signal.

A total of 37 samples with known *SOD2* polymorphism status were pyrosequenced. Pyrosequencing results showed 100% concordance compared to the consensus genotype (Table 3). In addition, four NTC samples were pyrosequenced.

### 3.4. Practical Results

Since going live in May 2018, we have sequenced 415 samples on approximately 120 different runs without any failures. We determined their genotypes to be 106 Ala/Ala (50.1%), 204 Ala/Val, and 105 Val/Val (49.9%). Controls never failed on any of the runs, and NTC was never contaminated/amplified.

Samples determined to have an Ala/Ala genotype were reported as homozygous wildtype for *SOD2*. In contrast, samples that showed the Ala/Val genotype were reported as heterozygous, and those determined to have the Val/Val genotype were reported as homozygous variants for *SOD2*. Reports cited that the Val/Val genotype has been associated with a modestly increased risk of cardiomyopathy in the Japanese population [28] and diabetic nephropathy [29] in general. Genetic counseling was recommended for consideration.

## 4. Discussion

We developed an assay for an ongoing clinical trial in a CLIA-certified laboratory that is routinely able to genotype samples for the *SOD2* p.A16V SNP. Analytical validation was performed on 37 samples used in the 1000 genomes project. The assay achieved 100% accuracy. Genotypes determined by the pyrosequencing assay demonstrated complete concordance with orthogonal assays (Sanger and Illumina sequencing) run on the same samples.

The validated assay has been used in a phase III, randomized, double-blind clinical trial (NCT03535675) and two ongoing trials in breast and prostate cancer (NCT04499950, NCT05471414). All samples are being received and processed in a timely manner. The outcomes of these trials will tell us more about the prognostic potential of this biomarker.

Men with Ala/Ala genotype in the *SOD2* gene (rs_4880) have a higher risk of aggressive prostate cancer in the presence of low antioxidant concentration^8^. Previous studies have demonstrated that this subgroup of men may benefit from compounds that reduce oxidative stress [8,30,31]. In this study, we present an analytical validation of the *SOD2* genotyping assay with outstanding analytical sensitivity, specificity, accuracy, and precision.

Further, this *SOD2* genotype has been implicated in various diseases, including prostate cancer [8,10,11,31,32,33,34,35]. Given the association of higher cancer risk with the AA-*SOD2* genotype, one might question why AA-*SOD2* BCR prostate cancer patients would benefit from MPX treatment. The answer lies in the Physicians’ Health Study finding of a 10-fold increase in the risk of aggressive prostate cancer from high to low quartiles of antioxidant status in men with the AA genotype (but not in men with the VA or VV genotypes) [8]. In addition, when given alone without potential drug interactions, these antioxidants do no harm and might have other modest but nevertheless beneficial health effects [30,36]. Furthermore, two studies reported that breast cancer risk was elevated among premenopausal women with the AA-*SOD2* genotype who had low consumption of dietary antioxidants [37,38]. This newly developed assay will allow us to test the hypothesis that AA-*SOD2* men will show a significant decrease in PSA slope when treated with MPX compared to placebo. If the trial shows that the AA-*SOD2* BCR subpopulation will benefit from ellagic acid supplementation, the results will encourage physicians to check patients’ *SOD2* genotypes to make recommendations about antioxidant supplementation. Negative results should put to rest the claim that MPX and ellagic acid as single oral agents are active in a clinically meaningful way in this patient population. Finally, the development of this assay will allow for further exploration of the predictive capacity of AA-*SOD2* to predict the response to antioxidants in other cancers, such as early-stage breast cancer.

## Figures and Tables

**Table 1:** Comparison of Sanger to Illumina sequencing.

		Illumina *		
		Ala/Ala	Ala/Val	Val/Val
Sanger	Ala/Ala	13	0	0
	Ala/Val	0	12	0
	Val/Val	0	0	12

* Illumina data were obtained from the publicly available 1000 genomes database.

**Table 2:** Analytic Precision.

Inter-run Reproducibility	**Sample**	**Known Genotype**	**Run 1**	**Run 2**
	NA07048	C/T	C/T (51–49%)	C/T (51–49%)
	NA18488	C/T	C/T (51–49%)	C/T (49–51%)
	NA18489	T/T	T/T (2–98%)	T/T (1–99%)
	NA18507	C/C	C/C (100–0%)	C/C (100–0%)
	GM12878	C/T	C/T (51–49%)	C/T (50–50%)
	NTC	-	No amplification	No amplification
Intra-run Reproducibility	NA18501	T/T	T/T (2–98%)	T/T (1–99%)
	NA18505	T/T	T/T (1–99%)	T/T (1–99%)
	NA18949	C/C	C/C (98–2%)	C/C (99–1%)
	NA18499	C/T	C/T (49–51%)	C/T (50–50%)

Allele frequencies of each allele are shown in parentheses.

**Table 3:** Comparison of pyrosequencing to Sanger and Illumina sequencing.

		Consensus Sanger/Illumina *		
		Ala/Ala	Ala/Val	Val/Val
Pyrosequencing	Ala/Ala	13	0	0
	Ala/Val	0	12	0
	Val/Val	0	0	12

* Consensus sequence from Table 1.

## Data Availability

Data are contained within this article or supplementary material.

## Supplementary Materials

The following supporting information can be downloaded at: https://www.mdpi.com/article/10.3390/mps6010004/s1, Table S1: Validation Samples.
